# Soaring over open waters: horizontal winds provide lift to soaring migrants in weak thermal conditions

**DOI:** 10.1186/s40462-023-00438-6

**Published:** 2023-12-09

**Authors:** J. Škrábal, Š. Krejčí, R. Raab, E. Sebastián-González, I. Literák

**Affiliations:** 1 Department of Biology and Wildlife Diseases, Faculty of Veterinary Hygiene and Ecology, University of Veterinary Sciences Brno, Palackého tř. 1946/1, 61242 Brno, Czech Republic; 2 TB Raab GmbH, Quadenstrasse 13, 2232 Deutsch-Wagram, Austria; 3 Department of Ecology, University of Alicante, 03690 Alicante, Spain

**Keywords:** *Milvus milvus*, Sea crossing, Barriers, Migration, Raptors, Red Kite

## Abstract

**Background:**

For soaring birds, the ability to benefit from variable airflow dynamics is crucial, especially while crossing natural barriers such as vast water bodies during migration. Soaring birds also take advantage of warm rising air, so-called thermals, that allow birds to ascend passively to higher altitudes with reduced energy costs. Although it is well known that soaring migrants generally benefit from supportive winds and thermals, the potential of uplifts and other weather factors enabling soaring behavior remains unsolved.

**Methods:**

In this study, we GPS-tracked 19 Red Kites, *Milvus milvus*, from the Central European population that crossed the Adriatic Sea on their autumn migration. Migratory tracks were annotated with weather data (wind support, side wind, temperature difference between air and surface—proxy for thermal uplift, cloud cover, and precipitation) to assess their effect on Red Kites' decisions and soaring performance along their migration across the Adriatic Sea and land.

**Results:**

Wind support affected the timing of crossing over the Adriatic Sea. We found that temperature differences and horizontal winds positively affected soaring sea movement by providing lift support in otherwise weak thermals. Furthermore, we found that the soaring patterns of tracked raptors were affected by the strength and direction of prevailing winds.

**Conclusion:**

Thanks to modern GPS–GSM telemetry devices and available data from online databases, we explored the effect of different weather variables on the occurrence of soaring behavior and soaring patterns of migratory raptors. We revealed how wind affected the soaring pattern and that tracked birds could soar in weak thermals by utilizing horizontal winds, thus reducing energy costs of active flapping flight over vast water bodies.

## Introduction

Wind is an important environmental factor affecting birds’ migratory route selection and overall performance during migration and barrier crossings [1–4]. Especially for soaring birds, the ability to benefit from variable airflow dynamics is crucial due to the higher energetic costs of powered flight [5]. Wind flowing in the same direction as the migrant (tailwind) subsidizes energy costs by supporting the bird in its movement. In contrast, winds of opposite (headwind) or perpendicular direction (side wind) might increase the energy costs of flight by reducing migrant speed or by drifting it away from its aimed direction [6, 7]. Soaring birds also take advantage of warm rising air, so-called thermals, that allow birds to ascend passively to higher altitudes with reduced energy costs [8, 9]. Although soaring migrants generally benefit from supportive wind and thermals [7, 8], how they respond to dynamic weather conditions over a sea remains unsolved.

The open sea is considered a significant migratory barrier for all terrestrial, particularly soaring, birds [10]. Over open water, winds tend to blow stronger than over land, as ocean and sea present a smooth surface that allows the wind to blow without interruption [11]. Dynamic weather conditions, strong winds, and the inability to regain energy create hazardous conditions for sea-crossing soaring migrants. Moreover, the occurrence of thermals that can provide energy-free lifts is considered to be relatively low at sea [12]. In recent studies, the temperature difference between surface and air (∆T) has been used as a proxy for the occurrence of thermals [1, 13]. Positive ΔT values correspond to a warmer surface than the air temperature, where a warmer surface heats the air that is pushed up by colder and denser air, creating an uplift. Negative values represent sinking air [14]. Soil particles generally have a lower thermal stability than water, meaning that the land surface is more efficiently heated or cooled than water. This different physical characteristic explains why thermals, which are essential for soaring migrants, are formed stronger on land than over sea [15]. The costs of sea crossings are reflected in studies of birds that fly along the coasts [16] or perform long detours on their migration and use shorter over-water passages to avoid long crossings of open seas [12, 17]. However, there is evidence that some soaring migrants soar in thermals that occur over the sea and thus reduce the energy costs of such crossing [18].

Studies that investigated the presence of uplift [13, 18–20] explored them as an important source of passive altitude gain in soaring migrants. Nourani et al. [7] found that weather conditions over open sea during different seasons can create more suitable migratory corridors for soaring migrants than those that occur over land. A few years later, using high-frequency GPS data, Duriez et al. [18] presented the first evidence of soaring behavior in raptors over the sea. This finding presented the idea that soaring migrants benefit from uplifts over a sea more than expected. However, Nourani et al. [13] conducted a study that found that sea-crossing soaring migrants prefer to select areas with prevailing wind support over areas with possible occurrence of uplifts. Although these studies brought precious information that built the foundation of modern understanding of how soaring migrants overcome open sea, they did not explore if the presence of soaring behavior is conditioned by the presence of uplifts per se [13, 18–20], mainly due to the lack of fine-scaled GPS data. Therefore, the potential of uplifts and other weather factors that can enable the soaring behavior of soaring migrants, allowing them to travel across natural barriers with lower risk and energy outcomes, merits further research.

Knowing how birds adapt their flights across migratory barriers remains an open and important question for understanding the evolution of migratory routes and sea-crossing strategies. In this paper, we try to answer this question by investigating fine-scaled GPS data obtained from Red Kites (*Milvus milvus*, a middle-size European raptor) that crossed the vast open waters of the Adriatic Sea (approximately 200 km) on their autumn migration from Central Europe to southern Italy. Red Kites from Central Europe winter in different Mediterranean countries of Europe [21]. Most birds from this population migrate through the continent, and only a small part cross over the Adriatic Sea [21], where the katabatic Bora wind blows in the southwestern direction [22]. These cold katabatic winds can be beneficial for crossing migrants not only by creating favorable wind support but also by bringing high-density air that decreases the frequency of energetically costly flapping flights [23] and thus can create reliable freeway for the small part of the Central European Red Kite population during autumn migrations [24].

Our study offers an opportunity to explore how soaring migrants, well adapted for flying in continental lowland and hilly areas, face the environmental conditions of the vast open sea and land terrain during their annual migratory movement. Here, we aim to determine (1) what weather conditions affected the timing of soaring migrants to overcome the Adriatic Sea, (2) what weather conditions enabled the soaring behavior of soaring migrants, and (3) how weather conditions affected soaring patterns over land and sea. The individual decision to engage in sea crossing and no evolutionary adaptations for life over sea makes Red Kites the perfect model species to study the plasticity of flight behavior in land-dwelling soaring raptors over open waters. Based on previous studies, we expect that wind support will play a significant role in the decision to initiate sea crossing [13, 16, 19]. We further assume the soaring behavior to be enabled primarily by the presence of thermals and potentially by horizontal winds [1].

## Material and methods

### Bird tagging and data collection

In this study, we explore the behavior of 19 Red Kites from the Central European breeding population (Austria, Czech Republic, and Slovakia) that crossed open waters of the Adriatic Sea during their autumn migration movements. Red Kites were fitted with telemetry loggers with solar panels as nestlings (20 g; Ecotone, Poland, www.ecotone.pl and Ornitela, Lithuania, www.ornitela.com). The average weight of tagged nestling was 950 ± 47 g, meaning that the logger represented approximately 2.1% of their body weight, under the recommended 3% threshold [25, 26].

Loggers were fitted onto the backs of the birds using harnesses (backpacks) consisting of a 6 mm Teflon ribbon encircling the body by two loops around the bases of the wings and joined in front of the breastbone. Loggers function in GPS (Global Position System)/GSM (Global System for Mobile Communication) systems. Ecotone loggers were set to collect one position fixed per 1–6 h, and Ornitela loggers were set to collect data in the range from 1 fix per 15 min to 1 h or in a 5-min burst (1 fix per second) followed by a 10–15-min pause to limit the battery drain. Accelerometer and magnetometer data (in g-force/1000 and milliGauss (mG), respectively) were collected only for Ornitela loggers in the same frequency as GPS positions. We used ArcGIS Pro (Esri, Redlands, CA, USA) to analyze the coordinates of bird positions and create migration maps. The raw datasets analysed during this study are available in Movebank Data Repository [27].

### Data processing, migration characteristics

We processed positional data (coordinates) for each bird individually. These data were separated into migration modules. The sea crossing was defined as the migration period between the first and last location above the water body.

Most individuals (n = 14) that reached the western seashore of Croatia rested by the shore for at least two days before crossing the Adriatic Sea (in 24 cases out of 38 crossings). Thus, to test if weather conditions affected the decision to cross, we compared weather data (see below for the specific variables used) from the days of resting by the shore and the day of crossing. The meteorological data for the resting days were annotated for the coordinates recorded from 6:00 to 13:00 (the time range when our birds initiated the crossing). For the crossing, we chose coordinates recorded from 6:00 until the bird's distance from the shore exceeded the arbitrarily chosen threshold of 10 km. We chose this threshold as some resting birds explored the area above the Adriatic Sea within this distance but did not depart. If more than one location per hour was obtained, we averaged the weather variables per hour. We obtained 324 records from resting days and 56 records for days of initiating the sea crossing.

Red Kites exhibited soaring and non-soaring behavior during both sea and land crossings. We followed the approach described by Williams et al*.* [28] and Duriez et al*.* [18] to sort the flight behavior into three categories of movement: soaring, gliding, and flapping.

The high resolution of the datasets made it easy to detect circling and ascending behavior, indicating thermal soaring. However, we used 3-axis accelerometer and magnetometer data for more systematic behavior classification and the sensor analysis software Framework4, available from https://framework4.swan.ac.uk/. Firstly, we omitted locations with recorded speeds lower than 1 km/h to eliminate roosting points. After that, we used the 3-axis accelerometer data to identify between active/flapping flight (strong oscillations in vertical z-axis) and passive flight (gliding/soaring—smooth in vertical z-axis between 800 and 1400 mG) [18, 28]. Once we selected the passive flight, we used the 3-axis magnetometer data to sort out thermal soaring (oscillations in the x-axis) and gliding (smooth x-axis) [18, 28]. After annotation, we projected the dataset in ArcGIS Pro and checked if the annotated behavior matched the projected data. For our purposes, we only used data from soaring and flapping flights. We did not include gliding flight because soaring migrants can glide in variable conditions once they reach the required altitude, regulating their airspeed and sink ratio [29], and we were interested in exploring suitable weather conditions for soaring over the flapping flight. After removing data annotated as gliding, we again projected the data in ArcGIS Pro. We chose only segments with more than 10 points in 1 s intervals with clear soaring or non-soaring patterns. We recorded in total 3 394 (7 807) and 1 757 (12 712) GPS positions in 1 s intervals for flapping and soaring flight over the sea (land), respectively. For each individual, we calculated the climb rate of burst collection as the difference between the segment's maximal and minimal heights above sea level. We observed three different soaring patterns over sea and land. We divided them into three groups by the trajectory shape: a) staircase—diameter of turns > 10 m, b) spiral—diameter of turns < 10 m, and c) s-shape—no full turns observed. Each GPS burst with recorded soaring behavior was categorized into these three groups. Few segments contained both staircase and spiral patterns. In this case, we annotated the segments by the most frequent pattern.

### Weather data

Weather data, such as u and v components of wind—vectors of speed and direction of the wind used to calculate wind support and side wind, air temperature, sea/land surface temperature, cloud cover, and total precipitation were obtained for each GPS position via the ENV-data track annotation service provided by Movebank [30] by ECMWF ERA5 reanalysis database with a temporal and spatial resolution of 1 h and 0.25°, respectively. We used the bilinear interpolation method for wind components and the nearest-neighbour method for temperature, precipitation, and cloud cover. Before extracting data, we explored the birds' altitude while crossing. Weather data of the sea crossings were extracted for each coordinate in real-time, and a pressure level of 1000 mb corresponded to an altitude of around 150 m. We extracted data separately for one individual at a pressure level of 925 mb as it flew over the sea at a much higher altitude. To assess the effect of weather on an individual's decisions to cross over the sea, we used the weather data from 100 m above the surface. As the birds migrated over land in higher altitudes, for this position, we obtained the weather data at a pressure level of 925 mb, corresponding to an altitude of around 760 m. Wind support and side wind were calculated by function NCEP.tailwind using RNCEP package [31], which calculates wind support and forward and sideways movement according to the equation of Tailwind (Tailwind = wind speed * cos (α), where α is the angle of the wind from the direction of travel). The azimuth of direction travel was measured in ArcGIS Pro. For sea crossing—between birds’ first and last location over the sea, and for land crossing—between breeding site and crossing point at Croatian coast. Equation Tailwind considers wind support as the flow component moving parallel to the specified direction (tailwind), with negative values indicating flows against the specified direction (headwind). We also calculated the temperature difference (ΔT) between surface and air as a meaningful proxy for uplift over water [13]. We calculated ΔT as the difference in temperature between the surface and the air in mean altitudes of birds’ flight (either 150 or 760 m).

### Statistical analysis

We performed the Mann–Whitney U test and Chi-square tests to test the differences between flight metrics during sea crossing and flying over land (wind support, side wind, temperature, altitude, and climb rate). Before any statistical analyses, we run the Shapiro–Wilk test to evaluate the normality of the data. To assess the effect of weather on an individual's decision to cross over a sea in relation to flow assistance, side wind (in absolute values), precipitation, temperature, and total cloud cover, we used a binomial generalized linear mixed model (GLMM) where the probability of departure was a binomial response variable. In these models, we included data from all 19 birds. To model the effect of weather on birds' movement across sea and land during autumn migration, we used another series of binomial GLMM models where the occurrence of soaring was a binomial response variable (soaring behavior = 1, flapping behavior = 0). In these models, only birds with telemetry loggers set to burst collection were included in the soaring models over sea and land (n_individuals_ = 4) due to the high frequency of coordinates recording. We analyzed the possible occurrence of soaring behavior in relation to wind support, side wind (in absolute values), cloud cover, and temperature difference (ΔT) between surface and air. We did not include precipitation in this model, as there was no precipitation during crossings. Firstly, we wanted to use all recorded locations with a frequency of 1 location per second. However, our models showed high autocorrelation of residuals. Therefore, we used one location per 30 s and averaged annotated meteorological data. Based on previous findings that sea crossing birds prioritized tailwind and areas with a high tailwind and ΔT [13], we fitted soaring models with interactions among these two variables. Two additional binomial GLMM models were used to test if weather conditions can predict soaring patterns. As we observed two soaring patterns over land and sea, we ran two models, one for each terrain, where the annotated soaring pattern was a binomial response. Again, we used averaged data in 30-s intervals to avoid high autocorrelation of residuals. In models regarding soaring vs. flapping behavior and soaring patterns over land, we observed a non-linear relation between wind support and the dependent variable. Therefore, we included polynomial terms for wind support in those models. In soaring models over sea, we observed a linear relation between wind support and the dependent variable. We explain this difference by absence of negative wind support (headwind) over a sea. We fitted our models in R software using the ‘lme4’ package [32]. We checked the dataset for multicollinearity and only used variables that were not highly correlated (r < 0.6). We omitted cloud cover from soaring models, as it was highly correlated with ΔT (r = 0.83). Before any analysis, we standardized the predictor variables using the 'scale' function to make the coefficients of our models comparable. We located outliers within independent variables and included them in our models. Weather conditions can reach extreme values; therefore, removing outliers could alter our study's aim of assessing the bird's response to changing weather conditions. In every model, an ID of birds was added as a random factor to control for variations in individuals. Model estimates were obtained by averaging the best-supported models with ΔAIC (Akaike information criterion) (soaring models) or ΔAICc (decision to cross model) lower than two, using the ‘dredge’ function in MuMIn package [33]. We calculated the predictive accuracy of each model using the k-fold-cross-validation method with 10 folds via the 'performance_accuracy' function in the ‘performance’ package [34]. All statistical tests were performed using an α value of 5%, and all mean values are presented (± standard deviation; SD) unless stated otherwise.

## Results

### The decision to cross the sea

**Fig. 1:**
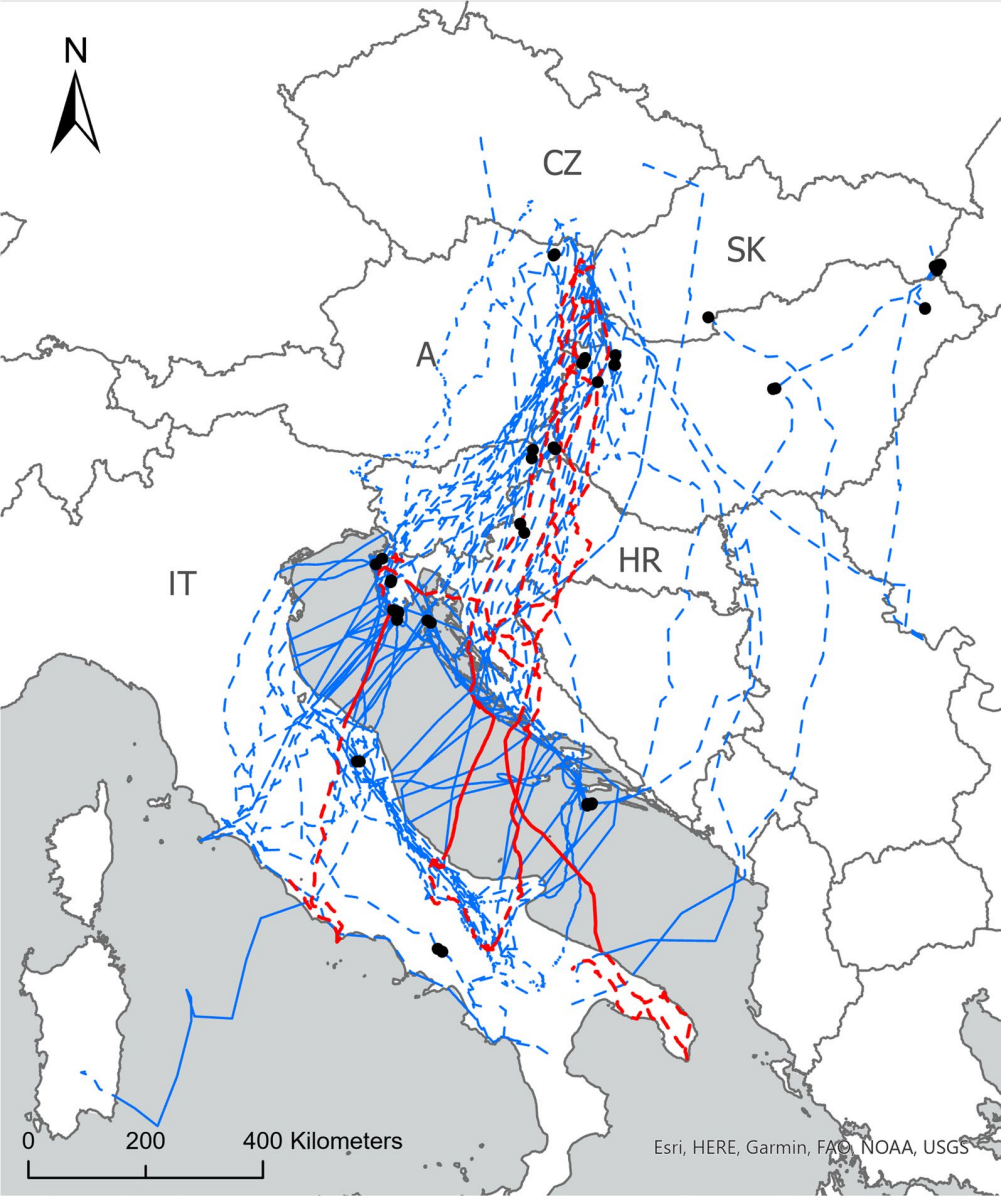
Map of autumn migratory movement of nineteen Red Kites from Central Europe that crossed the Adriatic Sea. Dashed lines depict flight above the continent and full lines of sea crossing. Red lines represent trajectory of four birds with 1 s data collection that were used in models comparing soaring and flapping flight. *A* Austria, *CZ* Czech Republic, *SK* Slovakia, *IT* Italy, *HR* Croatia

**Fig. 2:**
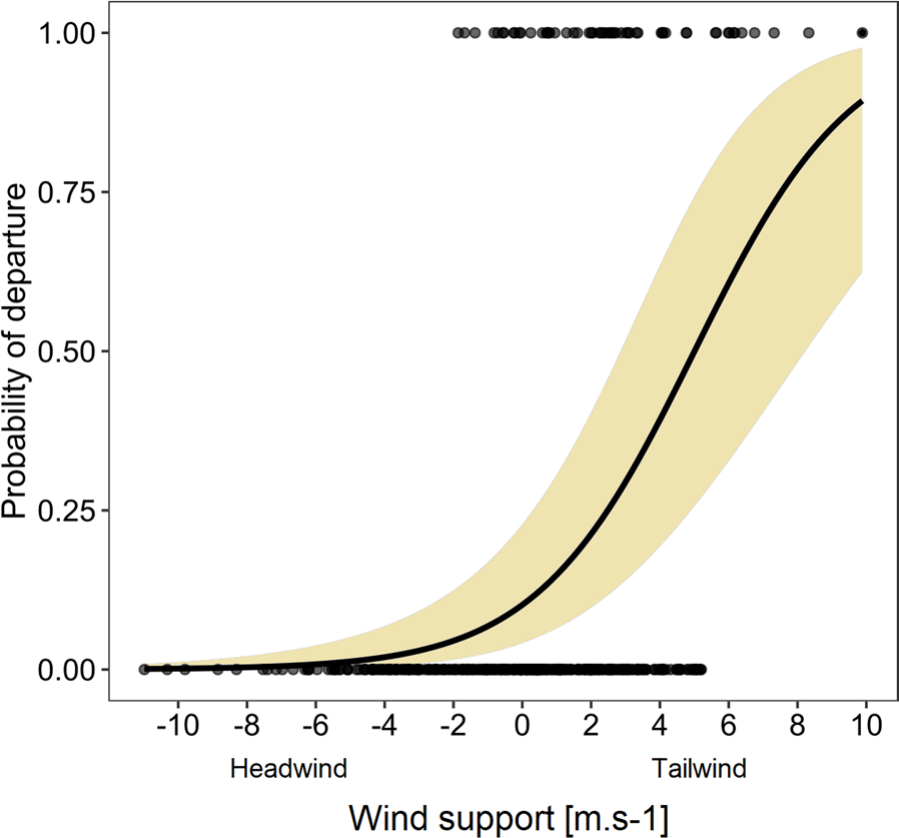
Predicted effect of a wind support on the probability of departure over Adriatic Sea with confident interval 95% of our best GLMM (generalized linear mixed model)

We examined the migration behavior of Red Kites from Central Europe that crossed the Adriatic Sea on their way to their wintering ground (Fig. 1). In total, we recorded 38 autumn sea-crossings that took place from September to November (Table 1). In order to explore what factors affected the decision to initiate a sea crossing, we examined the weather conditions experienced by the individuals who rested on the western Croatian coast before initiating the crossing over the Adriatic Sea. Results of the averaged model (accuracy 75%) showed that birds tended to initiate the crossing with a supportive wind (tailwind) and rested by the coast if the adverse winds prevailed (Table 2a, Fig. 2). Other factors included in the model did not have statistical support.

**Table 1:** Estimates of autumn crossing of Adriatic Sea by tagged Red Kites during 2015 and 2022

Migration component	N^a^	Autumn crossing Mean ± SD
Departure time	38 (19)	10:10 ± 3 h
Arrival time	38 (19)	13:35 ± 2.5 h
Departure date	38 (19)	03.10. ± 36 days
Average speed (km/h)	38 (19)	58 ± 13
Duration (hours)	38 (19)	3.2 ± 1 h
Route length (km)	38 (19)	189 ± 37

^a^Number of migration episodes (number of tagged individuals)

**Table 2:** Fixed effects on sea crossing probability, soaring probability and soaring patters

Model	Predictor	Estimate	Std. Error	z value	*p* value	P.A. %	R^2^_c_	R^2^_m_
(a) Crossing initiation	(Intercept)	− 1.97	0.46	4.26	0.00	75	0.52	0.31
	Wind support	0.99	0.27	4.38	0.00			
	Side wind	0.30	0.19	1.58	0.11			
	Cloud cover	− 0.07	0.20	0.38	0.70			
	Precipitation	− 0.47	0.30	1.57	0.12			
(b) Soaring over sea	(Intercept)	0.01	0.23	0.05	0.96	83	0.68	0.49
	Wind support	1.51	0.35	4.36	**0.00**			
	Side wind	1.65	0.48	3.44	**0.00**			
	ΔT	1.97	0.45	4.42	**0.00**			
	Wind support: ΔT	1.65	0.27	6.06	**0.00**			
(c) Soaring over land	(Intercept)	0.15	0.08	1.87	0.06	81	0.18	0.10
	poly(Wind support,2)1	0.01	0.09	0.07	0.94			
	poly(Wind support,2)2	− 0.27	0.10	− 2.68	**0.01**			
	Side wind	− 0.15	0.09	− 1.71	0.09			
	ΔT	0.35	0.09	3.85	**0.00**			
	Wind support: ΔT	− 0.15	0.13	− 1.19	0.23			
(d) Soaring pattern over sea	(Intercept)	− 2.79	0.77	− 3.60	**0.00**	80	0.72	0.54
	Wind support	3.42	1.47	2.31	**0.02**			
	Side wind	3.18	2.94	1.30	0.19			
	ΔT	3.82	2.61	1.21	0.22			
(e) Soaring pattern over land	(Intercept)	0.61	0.11	5.37	**0.00**	68	0.26	0.12
	poly(Wind support,2)1	− 0.60	0.13	− 4.61	**0.00**			
	poly(Wind support,2)2	− 0.80	0.14	− 5.63	**0.00**			
	Side wind	− 0.24	0.12	− 2.03	**0.04**			
	ΔT	0.14	0.12	1.19	0.28			

Fixed effects on sea crossing probability, soaring probability and soaring pattern as estimated by averaging our best GLMMs (ΔAIC or ΔAICc < 2) with individual ID included as a random factor (except d and e)

Significant results are highlighted in bold

*GLMM* generalized linear mixed model, *ID* identification number, *ΔT* difference between surface and air temperatures. *P.A.* predictive accuracy, *R*^*2*^_*c*_ conditional variance explained, *R*^*2*^_*m*_ marginal variance explained

### Flight behavior over the land and sea

Migrating birds spent more time flapping and less time soaring when flying across the sea than over land (Table 3). The ratio between GPS positions annotated as soaring and flapping flight was reversed above these two terrains. ΔT was more than four times larger at land, reflecting the lower occurrence of thermals over the sea (Table 3). Furthermore, the mean climb rate was twice higher at land (1.1 m s^−1^) than at sea (0.6 m s^−1^) (Table 3). Regarding the wind conditions, we found that Red Kites experience stronger wind support at sea during soaring and flapping flight (Table 3). In contrast to sea crossing, at land, migrating birds experienced mainly negative wind support (headwind) and stronger side wind during both flapping and soaring flight.

**Table 3:** Comparison of flight metrics during sea crossing and flying over land

	Migration over sea	Migration over land	Test
Soar/Flap ratio	1/1.9	1.6/1	
Wind support while soaring (m s^−1^)	4.8 ± 2.3	− 0.9 ± 4.5	U = 9593, ***p* < 0.05**
Wind support while flapping (m s^−1^)	2.7 ± 3.9	− 2.1 ± 4.6	U = 4032, ***p* < 0.05**
Side wind while soaring (m s^−1^)	2.5 ± 1.4	2.6 ± 1.8	U = 4334**, *p* < 0.05**
Side wind while flapping (m s^−1^)	1.9 ± 1.5	3.0 ± 2.1	U = 7355, ***p* < 0.05**
ΔT while soaring	1.1 ± 0.5	5.4 ± 1.8	U = 5793, ***p* < 0.05**
ΔT while flapping	1.2 ± 0.7	5.1 ± 2.0	U = 9012, ***p* < 0.05**
Mean altitude while soaring (m a.s.l.)	243 ± 215 (43–906)	738 ± 355 (63–2043)	U = 25,137, ***p* < 0.05**
Mean altitude while flapping (m a.s.l.)	101 ± 129 (20–978)	1051 ± 372 (247–2061)	U = 24,435, ***p* < 0.05**
Mean climb rate while soaring (m s^−1^)	0.6 ± 0.9 (0.1–1.5)	1.1 ± 0.6 (0.2–2.8)	U = 239, ***p* < 0.05**

Side wind speed is represented in absolute values. Soar/Flap ratio was calculated as a ratio between GPS positions annotated as soaring and flapping

Significant results are highlighted in bold. *ΔT* temperature difference between land and air

**Fig. 3:**
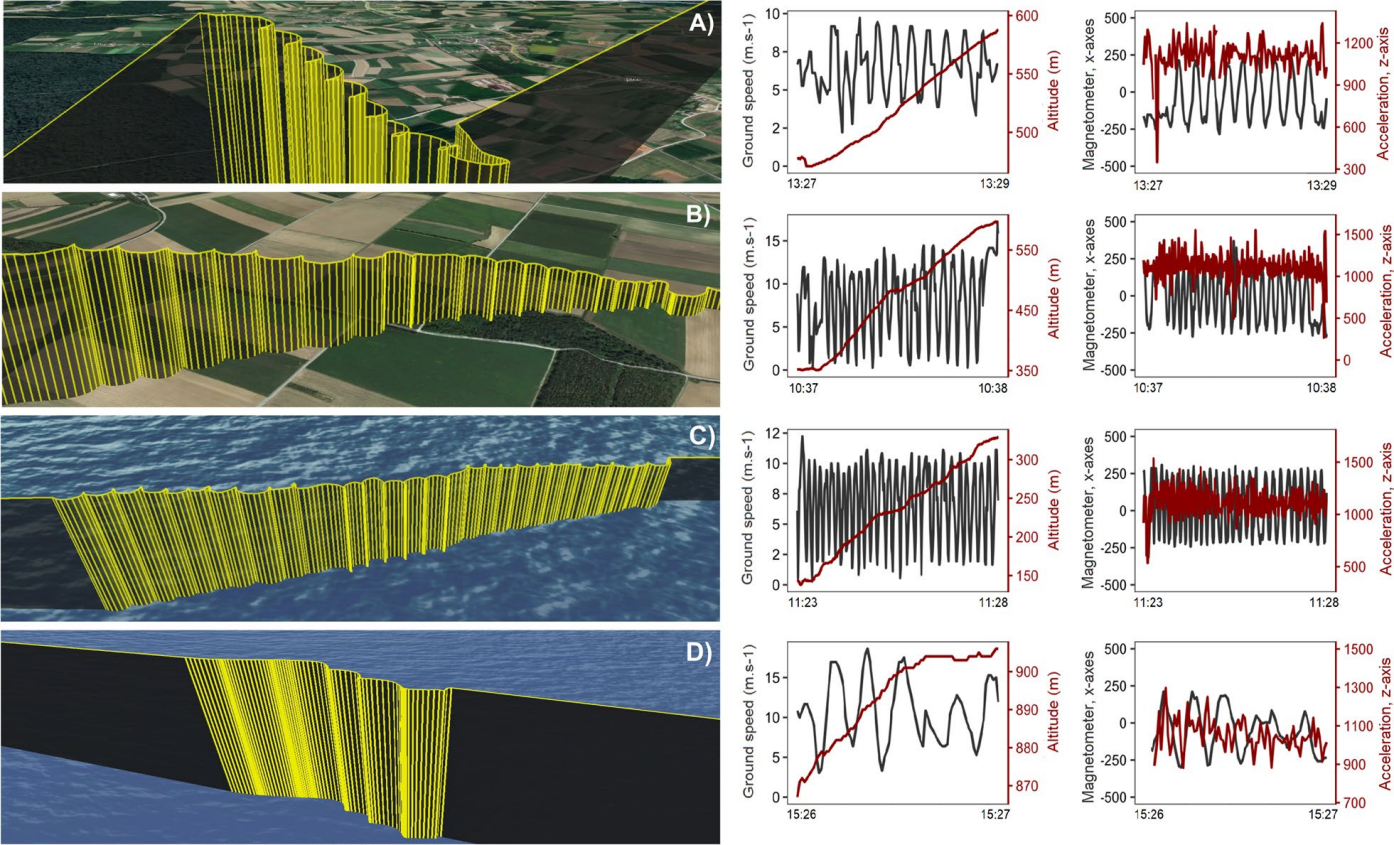
Three-dimensional view of soaring movement employed by Red Kites over the land (**A**, **B**) and Adriatic Sea (**C**, **D**). The GPS position was obtained in 5-min bursts of frequency, one location per second, followed by a 10-min break. Yellow vertical lines show the projection of the 3D track over the 2D surface. The panels in the middle show the differences in oscillation of birds' speed (m s^−1^) and gain in altitude. The panels on the right show the acceleration (*z*-axis, in black, mG) and the magnetometer (*x*-axis, in red, mGauss) signals. Oscillation on birds' speed and magnetometer reflect the circular movement

As we explored the soaring behavior of Red Kites, we observed variability in soaring patterns among birds (Fig. 3). Overland, birds employed two soaring patterns with either  staircase (8 288 GPS positions, Fig. 3A) or spiral (4 365 GPS positions, Fig. 3B) trajectory, depending on the strength of wind support and side wind (Table 2e). The stronger the wind support and side wind, the shorter the circular movement dimensions, the more horizontal the overall trajectory, and as a result the distance between turns increased. In weak to no wind support, birds soared in larger circular patterns, often overlapping each other.

**Fig. 4:**
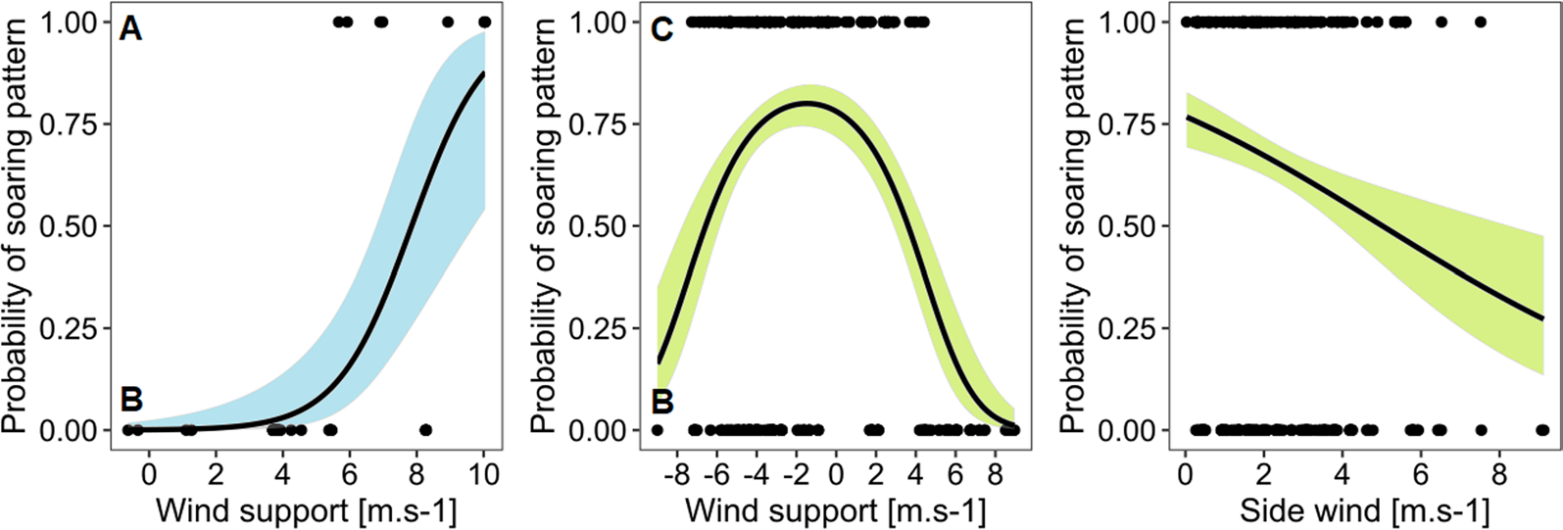
Predicted effects of statistically significant variables with confident interval 95% of our best GLMMs (generalized linear mixed models). Predicted effects of wind support and side wind on observed soaring patterns over sea (blue) and land (green). **A** s-shape pattern, **B** spiral pattern, **C** staircase pattern

Sea-crossing birds also employed two soaring patterns. The first pattern was observed in all birds, and its trajectory was identical to the spiral soaring pattern employed over land in the strong wind support and side wind (1 460 GPS positions) (Fig. 3C). However, unusual behavior was observed in three birds. One of them crossed the sea at higher altitudes (661 ± 230 m a.s.l.), while the others used typical altitudes in comparison to others (112 ± 90 m a.s.l.). These birds used a unique S-shaped soaring pattern (297 GPS positions) when they soared up, changing their airspeed without complete circulation movement (Fig. 3D). During this movement, birds experienced low ΔT (0.4 ± 0.3) and faced one of the strongest wind support (9.4 ± 1.1 m s^−1^) and side winds (3.1 ± 0.5 m s^−1^) from all sea-crossing Red Kites. Results of our model show that the wind support again conditioned the difference in occurrence between these two soaring patterns, but not side wind (Fig. 4, Table 2d).

**Fig. 5:**
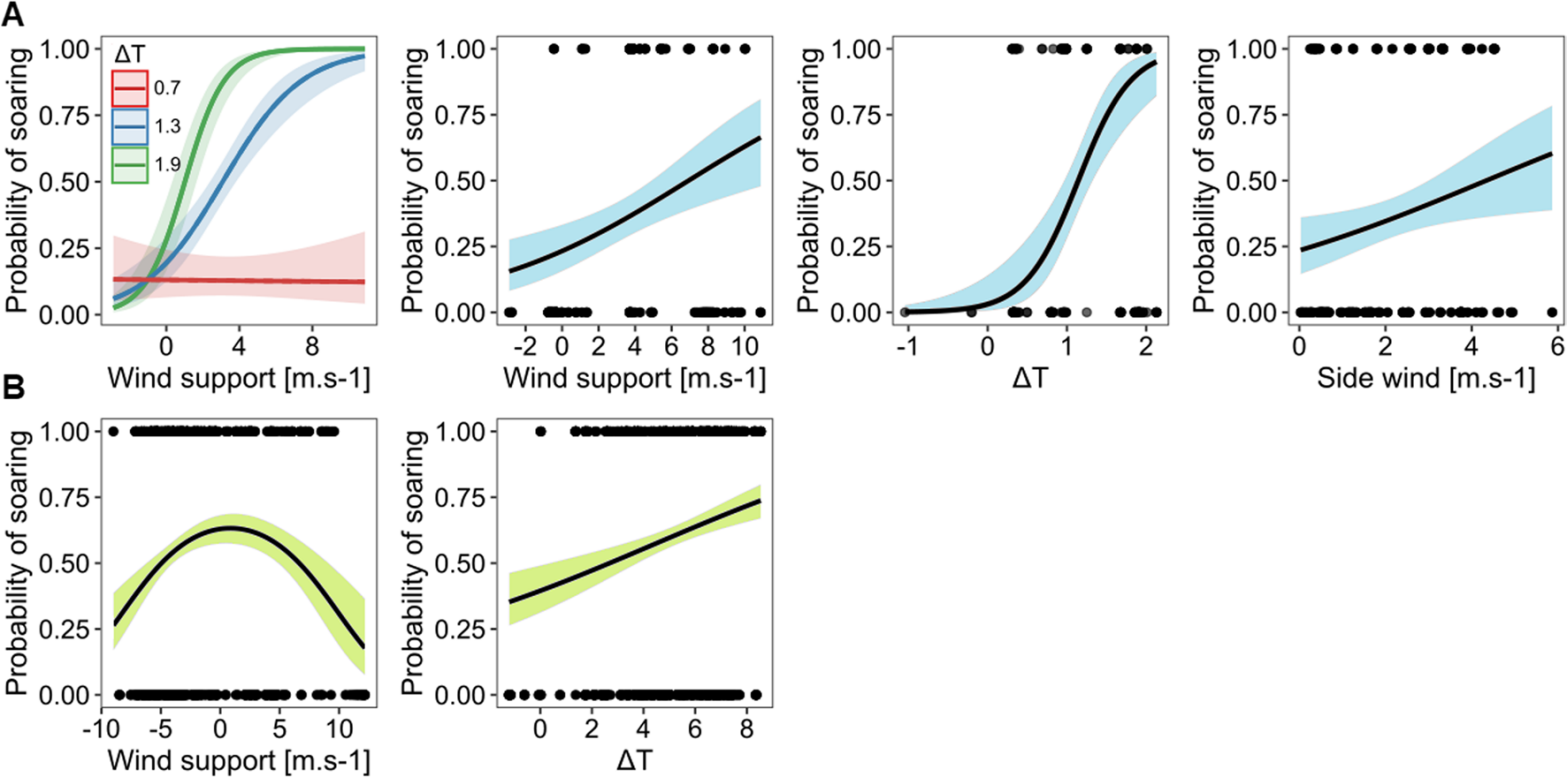
Predicted effects statistically significant variables with confident interval 95% of our best GLMMs (generalized linear mixed models). Predicted effects of wind support, side wind, ΔT and interaction between wind support and ΔT on the probability of soaring behaviour over the sea (**A**, blue) and over land (**B**, green). Negative values of wind support represent headwind and positive tailwind

When estimating what factors affected soaring behavior over land and sea, we found differences in wind utilization. During sea-crossing, the soaring behavior was affected by the presence of wind support, side wind, ΔT, and the interaction of wind support and ΔT (Table 2b, Fig. 5A). Our results showed that the stronger the wind support, ΔT, and side wind, the higher the probability of soaring behavior at sea. The best model, with a predictive accuracy of 83%, predicted a 50% probability of soaring at sea when the tailwind speed was 6 m s^−1^ and ΔT over 1° (Fig. 5A). Interaction between wind support and ΔT showed that rising values of both predictors increased the probability of soaring and that in low values of ΔT, the probability of soaring was small regardless of the wind support. The probability of soaring was also small while negative or weak wind support prevailed, regardless of the value of ΔT.

When flying over the land, wind support and ΔT had a significant effect on the probability of soaring (Table 2c, Fig. 5B). The highest probability of soaring was found with wind support between -5 to 6 m s^−1^, and outside this interval, the probability decreased. The effect of ΔT on the probability of soaring was smaller in comparison to the sea. The probability of soaring was 50% when the surface was approximately 3 °C warmer than the air at the altitude that birds flew in (model predictive accuracy 81%). Interaction between wind support and ΔT showed no effect on soaring over land.

## Discussion

Our study focused on soaring raptors’ abilities to cross over barriers with a particular interest in how weather conditions affected their soaring behavior over different terrains, as the ability to perform soar-glide flight is crucial for soaring migrants [5]. We found interesting results showing that wind utilization of soaring migrants varied above different terrains, that horizontal wind affected soaring patterns of tracked birds, and most importantly, that horizontal wind played an important role in inducing soaring behavior in weak thermals over open waters.

### Soaring behaviour

We found that the weather factors that affected the soaring performance of Red Kites differed while flying above open waters and land. Our model showed that wind support, side wind, ΔT and interaction of wind support and ΔT conditioned the presence of soaring behaviour over open waters. Although no studies are exploring the effect of weather factors on soaring behavior over a sea, Duriez et al. [18] reported that Ospreys *Pandion haliaetus* soared in thermal uplift while crossing over the open sea, which offered an idea that thermals are more frequent over the open seas than previously considered, and can play an important role for soaring migrants in surmounting open waters. However, higher occurrences of flapping flights, lower flying altitudes, and lower climb rates reported by our results indicate that their strength and abundance are lower in comparison to thermals occurring over land. Nourani et al. [13] found that soaring migrants were more affected by wind support than uplift while crossing overseas. Considering all the factors mentioned above, it is highly probable that while sea crossing, soaring migrants rely primarily on wind support rather than on the scarce occurrence of weak thermals to reduce the time and energy required for completing their risky journey over open waters [13, 19, 35].

By exploring the difference in soaring patterns observed above sea and land (Fig. 3), we found that the strength and direction of horizontal winds shaped soaring patterns. We observed that during spiral soaring, birds climbed through the air in the first quarter of the sharp turn with a short diameter followed by horizontal flight – creating a spiral-like trajectory caused by prevailing wind support and side wind [36, 37]. A similar trajectory was recorded in Ospreys, which used thermals for soaring up during sea-crossing [18]. This behavior connected with sharp turns is possible due to the ability of these birds to fly at higher bank angles, which considerably reduces their turning trajectory [38] and allows them to reduce the kinetic energy loss while turning into a headwind and use it for the climb. The ability to perform sharp turns can be crucial, especially for soaring migrants flying in the strong winds over the sea, because while circling, the birds need to turn back to a supportive wind before losing all their kinetic energy by the opposite wind in order to avoid an energetically costly flapping flight.

Three of the studied birds performed a unique pattern of soaring over the sea, where they moved in an s-shape, similar to the control flight observed in large soaring raptors [39]. We did not observe this soaring pattern over the land. Larger species of soaring raptors, such as Turkey Vultures *Cathartes aura*, follow a characteristically tortuous flight path when gliding at low altitudes, changing their vertical and horizontal direction while maintaining the same altitude. This behavior was explained by the use of shear-induced turbulence generated in areas where weather conditions are not optimal for the formation of thermals [39]. Although turbulences can explain this S-shaped soaring behavior, we observed an increase in altitude. Therefore, we do not think that birds used shear-induced turbulence when soaring over the sea. The three birds that performed this soaring pattern while facing strong wind conditions supposedly benefited from the strong wind support and side wind. We assume that these birds used their kinetic energy of movement gained by strong wind support to turn and climb up in the side wind (now headwind from the birds' perception), using the higher air pressure on the bottom of their wing as a force to push them up within short distances without the need of a complete circular movement, reducing energy costs [14]. We believe that this mechanism of exchanging kinetic energy to climb was employed in all soaring behavior over the sea with strong winds and explains the effect of wind support and side wind on the probability of soaring behavior over sea shown by our model. We assume that, in this manner, sea-crossing birds were able to soar up not only by using thermal uplift but also to horizontal winds in weak thermals in a way similar to dynamically soaring seabirds [40]. This assumption is supported by our results, which showed that without sufficient wind support, the probability of soaring was lower. Our finding provides the first evidence for the idea that soaring migrants have to utilize horizontal winds to gain elevation in weak thermals, as proposed by Duriez et al. [18]. Thus, the preference for strong wind support would not only reduce the time and, with it, connected energy costs [13, 19, 35] but also reduce the energy costs associated with frequent flapping flight.

Over the land, birds performed soaring behavior in the presence of thermals (high values of ΔT) and in low to moderate wind support. As the wind support increased, the probability of soaring decreased. Kites seemed to utilize this positive wind support to increase the distance travelled while flapping, over elevation gain via soaring. When we explored the trajectories of migrating Kites, we observed that birds often changed their heading and soared in the opposite direction. After soaring up, birds reoriented back to their intended direction and glided/flapped through the headwind that was prevailing over land during their autumn migration. Such behavior was reflected in our model, which predicted that individuals also soared in a headwind. Summarizing, Red Kites in this study showed great plasticity in response to changes in wind conditions along their migratory route [7, 29, 41].

### Initiating sea crossing

In autumn migration, Red Kites spent up to two weeks by the western coast of Croatia waiting for suitable winds to initiate the Adriatic Sea crossing. During this time, birds could choose a less risky route and fly through the continental part of northern Italy to reach their wintering destination. However, they decided to wait and cross the Adriatic Sea in a prevailing wind support. These birds waited for the Bora winds that blow from the direction of Croatia to Italy [22]. Because wind has a strong impact on flying costs [6], we suppose that such periodically occurring winds create annual freeways that can be utilized by Red Kites and other soaring migrants on autumn migration through the Adriatic Sea [13, 18, 24]. Our results support previous findings that showed the importance of supporting winds during migration and initiating departures [7, 20, 42]. By using this freeway, birds could benefit from supportive wind and reach the wintering destination in southern Italy with less energy than by detouring over the continent, at the expense of a possible later arrival to the wintering ground. A similar phenomenon was observed on the Oriental Honey-buzzard *Pernis ptilorhynchus* [19]. Suitable wind conditions on autumn migration allowed these soaring raptors to perform long crossings over the East China Sea and shorten their way to wintering ground [19].

## Conclusion

In this study, we explored the effect of different weather factors on the soaring behavior of soaring raptors, and we concluded that, although the principles of birds soaring in thermals have been recently studied [38, 42, 43], there are still some knowledge gaps on weak-thermal soaring behaviors that are essential for understanding birds’ migratory capabilities. We found wind support to be a key factor in the initiation of sea crossing and, surprisingly, along with side wind, in the occurrence of soaring behavior over sea. Birds were able to soar in weak thermals by utilizing horizontal winds, thus reducing the energy costs of active flapping during the crossing. Red Kites, as a model species of land-dwelling soaring migrants, showed great plasticity in utilizing winds over sea and land. By exploring the effect of different weather variables on the occurrence of soaring behavior and soaring patterns, this study brings another piece to the puzzle regarding the ability and capability of soaring raptors to cross over the open sea.

## Data Availability

The datasets analysed during the current study are available in the Movebank Data Repository, https://doi.org/10.5441/001/1.300 (Škrábal et al. 2023).

## References

[1] Literák I, Škrábal J, Karyakin IV, Andreyenkova NG, Vazhov SV (2022). Black Kites on a flyway between Western Siberia and the Indian Subcontinent. Sci Rep.

[2] Sergio F, Barbosa JM, Tanferna A, Silva R, Blas J, Hiraldo F (2022). Compensation for wind drift during raptor migration improves with age through mortality selection. Nat Ecol Evol.

[3] Vansteelant WMG, Shamoun-Baranes J, van Manen W, van Diermen J, Bouten W (2017). Seasonal detours by soaring migrants shaped by wind regimes along the East Atlantic Flyway. J Anim Ecol.

[4] Nourani E, Safi K, Yamaguchi NM, Higuchi H (2018). Raptor migration in an oceanic flyway: wind and geography shape the migratory route of grey-faced buzzards in East Asia. R Soc Open Sci.

[5] Bohrer G, Brandes D, Mandel JT, Bildstein KL, Miller TA, Lanzone M (2012). Estimating updraft velocity components over large spatial scales: contrasting migration strategies of golden eagles and turkey vultures: contrasting migration strategies of two raptors. Ecol Lett.

[6] Liechti F (2006). Birds: blowin’ by the wind?. J Ornithol.

[7] Becciu P, Panuccio M, Catoni C, Dell’Omo G, Sapir N (2018). Contrasting aspects of tailwinds and asymmetrical response to crosswinds in soaring migrants. Behav Ecol Sociobiol.

[8] Santos CD, Hanssemn F, Muñoz AR, Onrubia A, Wikelski M, May R (2017). Match between soaring modes of black kites and the fine-scale distribution of updrafts. Sci Rep.

[9] Williams HJ, Shepard ELC, Holton MD, Alarcón PAE, Wilson RP, Lambertucci SA (2020). Physical limits of flight performance in the heaviest soaring bird. Proc Natl Acad Sci.

[10] Mellone U (2020). Sea crossing as a major determinant for the evolution of migratory strategies in soaring birds. J Anim Ecol.

[11] Liu WT, Tang W, Xie X (2008). Wind power distribution over the ocean. Geophys Res Lett.

[12] Newton I (2008). The migration ecology of birds.

[13] Nourani E, Bohrer G, Becciu P, Bierregaard RO, Duriez O, Figuerola J (2021). The interplay of wind and uplift facilitates over-water flight in facultative soaring birds. Proc R Soc B Biol Sci.

[14] Pilot’s handbook of aeronautical knowledge 2016. Oklahoma City, OK: United States Department of Transportation, Federal Aviation Administration, Airman Testing Standards Branch; 2016.

[15] Mohamed A, Taylor GK, Watkins S, Windsor SP (2022). Opportunistic soaring by birds suggests new opportunities for atmospheric energy harvesting by flying robots. J R Soc Interface.

[16] Becciu P, Rotics S, Horvitz N, Kaatz M, Fiedler W, Zurell D (2020). Causes and consequences of facultative sea crossing in a soaring migrant. Funct Ecol.

[17] Mellone U, Limiñana R, Mallia E, Urios V (2011). Extremely detoured migration in an inexperienced bird: interplay of transport costs and social interactions. J Avian Biol.

[18] Duriez O, Peron G, Gremillet D, Sforzi A, Monti F (2018). Migrating ospreys use thermal uplift over the open sea. Biol Lett.

[19] Nourani E, Yamaguchi NM, Manda A, Higuchi H (2016). Wind conditions facilitate the seasonal water-crossing behaviour of Oriental Honey-buzzards *Pernis ptilorhynchus* over the East China Sea. Ibis.

[20] Santos CD, Silva JP, Muñoz AR, Onrubia A, Wikelski M (2020). The gateway to Africa: What determines sea crossing performance of a migratory soaring birds at the Strait of Gibraltar. J Anim Ecol.

[21] Literák I, Raab R, Škrábal J, Vyhnal S, Dostál M (2022). Dispersal and philopatry in Central European Red Kites *Milvus milvus*. J. Ornithol..

[22] Signell RP, Chiggiato J, Horstmann J, Doyle JD, Pullen J, Askari F (2010). High-resolution mapping of Bora winds in the northern Adriatic Sea using synthetic aperture radar. J Geophys Res.

[23] Schmaljohann H, Liechti F (2009). Adjustments of wingbeat frequency and air speed to air density in free-flying migratory birds. J Exp Biol.

[24] Kranstauber B, Weinzierl R, Wikelski M, Safi K (2015). Global aerial flyways allow efficient travelling. Ecol Lett.

[25] Phillips RA, Xavier JC, Croxall JP (2003). Effects of satellite transmitters on albatrosses and petrels. Auk.

[26] Puehringer-Sturmayr V, Loretto MCA, Hemetsberger J, Czerny T, Gschwandegger J, Leitsberger M (2020). Effects of bio-loggers on behaviour and corticosterone metabolites of Northern Bald Ibises (*Geronticus eremita*) in the field and in captivity. Anim Biotelemetry.

[27] Škrábal J, Literák I, Raab R. 2023. Study "Milvus_milvus_Soaring_over_Adriatic_sea". Movebank Data Repository. 10.5441/001/1.300.

[28] Williams HJ, Holton MD, Shepard ELC, Largey N, Norman B, Ryan PG (2017). Identification of animal movement patterns using tri-axial magnetometry. Mov Ecol.

[29] Vansteelant WMG, Shamoun-Baranes J, McLaren J, van Diermen J, Bouten W (2017). Soaring across continents: decision-making of a soaring migrant under changing atmospheric conditions along an entire flyway. J Avian Biol.

[30] Dodge S, Bohrer G, Weinzierl R, Davidson SC, Kays R, Douglas D (2013). The environmental-data automated track annotation (Env-DATA) system: linking animal tracks with environmental data. Mov Ecol.

[31] Kemp MU, Emiel van Loon E, Shamoun-Baranes J, Bouten W (2012). RNCEP: global weather and climate data at your fingertips: *RNCEP*. Methods Ecol Evol.

[32] Bates D, Maechler M, Bolker B, Walker S (2015). Fitting linear mixed-effects models using lme4. J Stat Softw.

[33] Bartón K. MuMIn: Multi-Model Inference. R package version 1.43.17. 2020. Available from: https://CRAN.R-project.org/package=MuMIn

[34] Lüdecke D, Ben-Shachar M, Patil I, Waggoner P, Makowski D (2021). performance: an R package for assessment, comparison and testing of statistical models. J Open Source Softw.

[35] Mitchell GW, Woodworth BK, Taylor PD, Norris DR (2015). Automated telemetry reveals age specific differences in flight duration and speed are driven by wind conditions in a migratory songbird. Mov Ecol.

[36] Weinzierl R, Bohrer G, Kranstauber B, Fiedler W, Wikelski M, Flack A (2016). Wind estimation based on thermal soaring of birds. Ecol Evol.

[37] Sage E, Bouten W, van Dijk W, Camphuysen KCJ, Shamoun-Baranes J (2022). Built up areas in a wet landscape are stepping stones for soaring flight in a seabird. Sci Total Environ.

[38] Ajanic E, Feroskhan M, Wüest V, Floreano D (2022). Sharp turning maneuvers with avian-inspired wing and tail morphing. Commun Eng.

[39] Mallon JM, Bildstein KL, Katzner TE (2016). In-flight turbulence benefits soaring birds. Auk.

[40] Richardson PL, Wakefield ED, Phillips RA (2018). Flight speed and performance of the wandering albatross with respect to wind. Mov Ecol.

[41] Klaassen RHG, Hake M, Strandberg R, Alerstam T (2011). Geographical and temporal flexibility in the response to crosswinds by migrating raptors. Proc R Soc B Biol Sci.

[42] Åkesson S, Hedenström A (2000). Wind selectivity of migratory flight departures in birds. Behav Ecol Sociobiol.

[43] Harel R, Horvitz N, Nathan R (2016). Adult vultures outperform juveniles in challenging thermal soaring conditions. Sci Rep.

[44] Percie du Sert N, Hurst V, Ahluwalia A, Alam S, Avey MT, Baker M (2020). The ARRIVE guidelines 20: updated guidelines for reporting animal research. PLOS Biol.

